# Indirect air CO_2_ capture and refinement based on OTEC seawater outgassing

**DOI:** 10.1016/j.isci.2021.102754

**Published:** 2021-06-19

**Authors:** Paul J.T. Straatman, Wilfried G.J.H.M. van Sark

**Affiliations:** 1 Indorama Ventures Europe B.V., Markweg 201, Harbour number 63473198, NB Europoort, the Netherlands; 2 Utrecht University, Copernicus Institute of Sustainable Development, Princetonlaan 8a, 3584 CB Utrecht, the Netherlands

**Keywords:** Earth sciences, Energy engineering, Energy resources, Energy sustainability, Engineering

## Abstract

In this paper, we propose a low-cost method to capture and purify CO_2_ from ocean water indirectly from the atmosphere. Atmospheric CO_2_ dissolves in seawater following Henry's law. In open-cycle ocean thermal energy conversion (OC-OTEC), being a heat engine, large quantities of water are used to generate electricity from temperature differences in the ocean. CO_2_ and other gases dissolved in seawater are extracted from seawater by a vacuum compressor, being essential for heat transfer in OC-OTEC. Non-condensable gases containing CO_2_ from OC-OTEC are currently considered a waste gas stream. Based on literature, we calculate cold water non-condensables containing up to 14% CO_2_. These non-condensables can be refined further to 80–90% purity with a water absorption process, inspired by those as used in the biogas industry. Levelized CO_2_ capture and purification costs were estimated to be 15–35 euro per ton, as only refinement costs are allocated of a waste gas stream of OC-OTEC.

## Highlights

• Indirect atmospheric CO_2_ capture from main power generation process in OTEC
• CO_2_ from OTEC purified with energy optimized water scrubber
• OTEC CO_2_-associated production costs in the range of 15–35 euro per ton

## Graphical abstract

**Figure:**
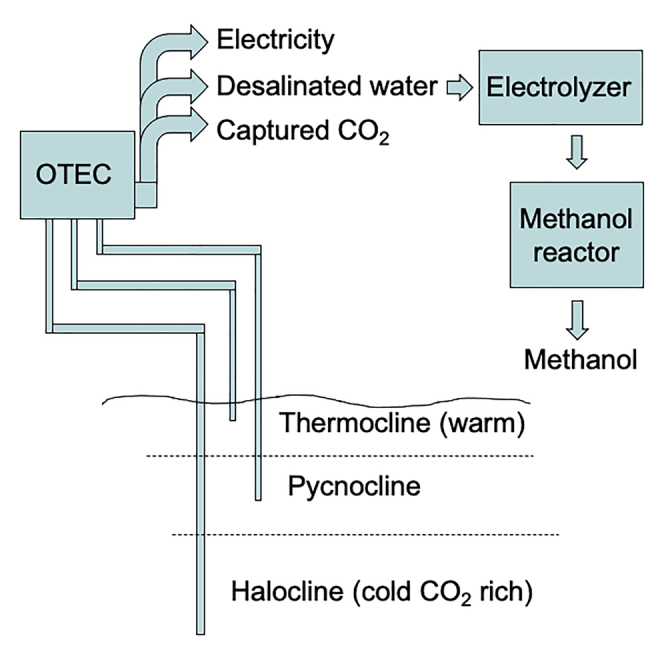

## Introduction

The effects of anthropogenic climate change have led to various international treaties that commonly aim to realize a society that is not emitting greenhouse gases anymore, in particular CO_2_. To this end, most focus is on massive deployment of (near-) zero emission energy conversion technologies (Ram et al., 2019). Alternatively, CO_2_ emission reduction by direct capture, either from non-zero emission energy conversion technologies, known as carbon capture and storage (Rackley, 2017), or directly from air is being investigated (IEA, 2020). In addition, a large resource of CO_2_ is found in Earth's oceans; 39,000 GtC (gigatonnes of carbon) currently reside in the oceans while only 750 GtC are in the atmosphere (Rackley 2017). Capture, partial utilization, and partial storage of that CO_2_ would reduce its concentration in air considerably as well. Although in the last decades several attempts have been made to realize technologies for capture of CO_2_ from ocean water (membrane filtration, electro dialysis (Eisaman, 2020)), referred to as indirect ocean capture (IOC), and from the atmosphere directly (direct air capture, DAC) (IEA, 2020), they all have been very costly and energy intensive, to run, to build, and to operate. Capture costs of CO_2_ with atmospheric capture vary between 100 and 600 US$ per ton of captured CO_2_ (IEA, 2020). A recent study detailed various cost aspects related to IOC and DAC cost potentials (Eisaman, 2020) and used a range of 10–200 US$ per ton.

In this paper, using an existing technology as a starting point, we propose a low-cost method for carbon capture from ocean water. This method is derived from analysis of open-cycle ocean thermal energy conversion (OC-OTEC) technology principles. We describe the capture principle by combining ocean thermal energy conversion (OTEC) and barometric vacuum desalination. We envisage that the resulting purified CO_2_ can be used either for sequestration and/or as a resource for synthetic fuel, if combined with hydrogen to form methanol or via a Fischer-Tropsch process to kerosene (Terwel and Kerkhoven, 2018). Rau and Baird (2018) and Eisaman (2020) describe the production of a synthetic fuel, methanol, directly from hydrogen, electrolyzed from water, and CO_2_, extracted from the environment. The price of such a fuel is highly dependent on the production cost of hydrogen and CO_2_. It should be noted that the CO_2_ capture method presented in this paper is highly dependent on the competitiveness of OTEC as such because the existence of OTEC is a prerequisite for realization of the proposed technology. In this paper, we focus on a low-cost CO_2_ production method to create an economic driver for the utilization potential of sequestration and use of synthetic fuel.

This paper is further organized as follows. We first review literature on CO_2_ extraction methods from air and ocean water and describe in detail CO_2_ release from OTEC operation. Then, we describe calculations based on literature data on the composition and quantities of CO_2_ that are emitted from the exhaust compressor with OC-OTEC. After that, we perform an economic analysis of the optional purification for the CO_2_ extraction process, followed by a discussion on further research. We close the paper with a conclusion and reflect on the limitations of this study.

## Results and discussion

### Atmospheric CO_2_ capture: Proposed methods

According to the International Energy Agency (IEA), there are currently 15 direct air capture plants in operation globally that together capture over 9,000 t CO_2_/year. The basic underlying principle of these technologies is the absorption and desorption of CO_2_ by a recycling working fluid that is selective for CO_2_ (IEA, 2020).

By 2030, direct air capture is estimated to reach almost 10 Mt CO_2_/year in IEA's Sustainable Development Scenario, but it will require large-scale demonstrations mostly to reduce costs (IEA, 2020). While the environmental benefits are obvious, direct capture technologies presently are not commercially attractive and need to be developed and optimized to increase their attractiveness. Prices of 100 euro per ton for atmospheric capture and 45 euro per ton for end of pipe capture (van Leeuwen, 2020) are common.

Fasihi et al. (2019) gave an extensive literature review and technoeconomic assessment of DAC. By 2050, DAC needs to be deployed on a large scale, to meet the Paris agreement, even when fossil fuel use will be mitigated (Fasihi et al., 2019). It was estimated that the need for CO_2_ capture will increase in the years 2020, 2030, 2040, and 2050 to 3, 470, 4,798, and 15,402 MtCO_2_/a, respectively. The concluded costs of estimated air capture in the year 2020–2050 were 815–199 euro per ton, respectively, in a conservative scenario. These capture costs would decrease under a capex learning curve with 15% learning rate to 79 euro per ton in the year 2050 (Fasihi et al., 2019).

### Oceanic CO_2_

Capture of oceanic dissolved CO_2_ is an indirect form of atmospheric capture because the ocean absorbs atmospheric CO_2_ (Eisaman, 2020). It is estimated that worldwide CO_2_ is absorbed from the air by the ocean at a rate of 300 tons per second (Huelsenbeck, 2013). Following Henry's law (Henry, 1803), all gases in the atmosphere including CO_2_, will dissolve in water. This process of dissolution is an equilibrium reaction. Ocean water contains CO_2_ in the form of dissolved gaseous CO_2_ and HCO_3_^-^ and CO_3_^2−^ due to reaction of CO_2_ with water. These concentrations are referred to as total inorganic carbon (TIC), based on concentration analysis using mass spectrometry (Murray et al., 2013), and depend on water temperature. Adding CO_2_ to seawater acidifies it, thus lowering its pH.

The extent of the equilibrium described by Henry's law depends on the kind of gas, temperature, pressure, and molar fraction of each component in the gas phase. Henry's law describes this characteristic dissolving behavior as follows:

(Equation 1) c=HP

Here, c is the concentration of the compound in water in mol/m^3^, P is the partial pressure of the compound in the gas phase in Pa, and H is the Henry volatility in mol/(m^3^·Pa), or, equivalently in M/atm.

Table 1 shows how Henry's law is applied to the ocean-earth atmosphere system in finding the concentrations of each gas component in water from their atmospheric concentrations. Note that these data are pressure, temperature, and composition dependent. For instance, under same temperature and pressure circumstances, the composition of dissolved gases may deviate significantly for seawater from pure water.

**Table 1:** Concentrations of atmospheric gases dissolved in water

Component	Molar fraction in air (−)	Henry volatility (mol/m^3^·Pa)	Concentration in water (mol/m^3^)
Nitrogen	0.78	1639	0.48
Oxygen	0.21	769	0.27
Argon	0.01	714	0.01
CO_2_	0.0004	29	0.01
Total	1	–	0.77

CO_2_ has a concentration of 400 ppm (or a fraction of 0.0004) in air.

The molar fraction of CO_2_ concerns only the molecular carbon dissolved. TIC is much larger at 2 mol/m^3^ as reported using chemical analyses on dissolved gases in seawater (Sverdrup, 1942). Also, a concentration gradient can be observed. Dore et al. (2009) report on the amount of dissolved inorganic carbon and pH as a function of depth down to 5 km below sea level. The concentration of dissolved inorganic carbon changes from 2.0 to 2.3 mol/m^3^ in the first subsurface km and remains about constant down to a depth of 5 km, while pH changes from about 8.1 to 7.8. The downstream effects of vacuum degassing are described by Green and Guenther (1990) by the following reactions. In order to keep the anoxic effects from the organisms that may be affected by it, subsurface discharge of 50 meters below sea level is part of the design.

Green and Guenther (1990) describe the following reactions of reversible dissolving and degassing of CO_2_ in water.

(Equation 2) CO_2_ (g) + H_2_O ⇆ CO_2_ (aq) + H_2_O

(Equation 3) CO_2_ (aq) + H_2_O ⇆ H_2_CO_3_

(Equation 4) H_2_CO_3_ ⇆ HCO_3_^-^ + H_3_O^+^

(Equation 5) HCO_3_^-^ ⇆ CO_3_^2−^ + H_3_O^+^

Reaction 1 is readily releasing CO_2_ (g) in sub atmospheric pressures. The loss of dissolved CO_2_ creates non-equilibrium where reactions 2, 3, and 4 compensate (Green and Guenther, 1990). According to Riley and Chester (1971), the half-life time of reaction 2 is in the order of minutes. This indicates that only a small fraction of the bicarbonate ions will be converted to CO_2_ (aq) during the short residence time of several seconds in a vacuum compartment in an OTEC installation. Effects on pH will be small but will force pH to go up in the range of 7.80–7.85. This pH change was also observed in an experimental setting, where we measured pH before and after vacuum degassing with a residence time of several seconds. This experiment was performed on a lab scale. The seawater samples were taken at the surface at the Dutch coastline. Furthermore, Green and Guenther (1990) measured salinity and partial pressure of CO_2_ before and after vacuum. The results are summarized in Table 2.

**Table 2:** Partial pressures and salinities of the cold water stream measured and calculated before and after vacuum degassing in an OTEC plant

Source of sample	PCO_2_ (ppm)	Measured salinity (ppt)	Calculated salinity (ppt)	TCO_2_ (mmol/m^3^)
Condenser upcomer	1075	34.307	34.402	2308.6
Condenser downcomer	925.3	33.985	34.003	2228.3

### Oceanic CO_2_ capture: Electrodialysis

Electrodialysis (Patterson et al., 2019; Eisaman et al., 2012) is a technology that can be used to extract CO_2_ from the ocean. It is based on the filtering principle of membranes combined with ionization to separate carbon dioxide from water (Eisaman and Littau, 2017). This requires energy, and Eisaman et al. (2012) report an energy intensity of 272 kJ per mol extracted CO_2_ for this technology. Energy costs per ton CO_2_ can be calculated to be 71 euro per ton, based on an assumed energy price of 50 euro per MWh.

### CO_2_ capture from OC-OTEC

OTEC (Masutani and Takahashi 2019) is a heat engine generating electricity by exploiting the limited temperature differences between the surface and deep ocean water layers. Deep in the ocean, at about one kilometer of depth, the temperature is generally about 0–4°C. At the surface, the temperatures are higher (>20°C) due to solar irradiation and show diurnal variations of a few degrees. Therefore, OTEC is generally considered as a form of solar energy, where the ocean surface acts both as a collector and thermal energy storage in one. While thermodynamic (Carnot) efficiency is rather low due to the small temperature differences and large quantities of water needed for the operation of OTEC, solar irradiation is converted to electricity.

**Figure 1:**
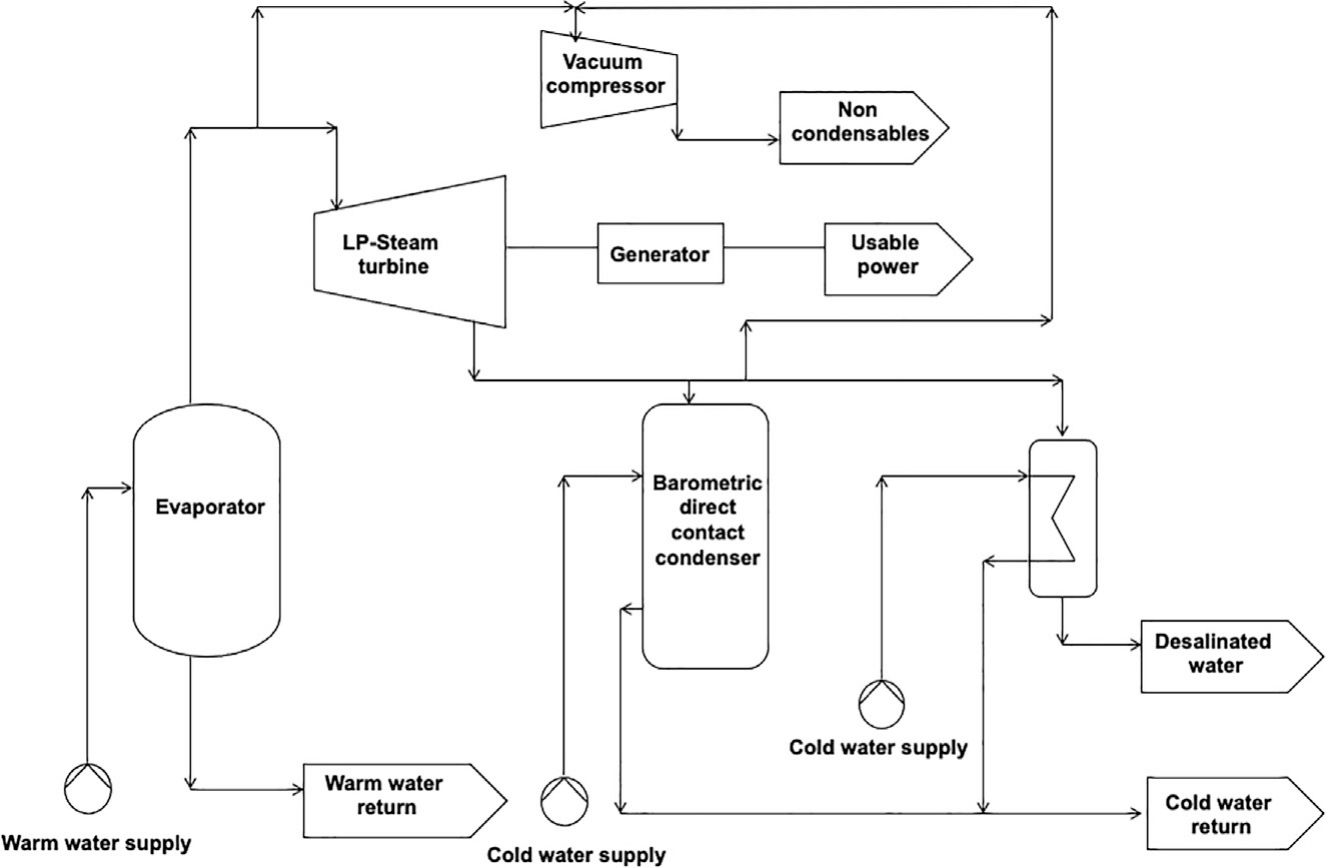
Schematic drawing after a scheme by Vega (1999) of an open-cycle OTEC/barometric desalination system, and the waste gas stream of non-condensables, containing CO_2_ The power scale of this figure is referred as generally 1 megawatt, and all flows and energy flows in the text are derived from this installed power.

In Figure 1, a schematic overview of a typical OC-OTEC system is presented, made after a process flow diagram and mass balance of an OC-OTEC with integrated barometric desalination reported by Vega (1999). Warm surface water is used to evaporate a working medium. In the case of OC-OTEC, this is the seawater that flashes under vacuum. The vapor drives a turbine generator. The vapor is thereafter condensed by cooling with cold ocean water from the deep.

In an OC-OTEC, the water only evaporates when the air is removed. The water therefore flows through a vacuumed compartment. Because the pressure on the water is suddenly decreased to almost vacuum, dissolved gases will evaporate out of the fluid. Among oxygen and nitrogen, CO_2_ makes a large part of this. These gases accumulate in the vacuum chamber of a condenser and evaporator of OTEC installations and are extracted by the vacuum pump and released into the atmosphere or mixed with one of the discharge water streams to prevent emission to the atmosphere. Green and Guenther (1990) have investigated emissions from OC-OTEC and found that CO_2_ emissions are 11.7 and 26.8 gram CO_2_ per kWh generated for warm and cold seawater, respectively, thus totaling 38.5 g CO_2_/kWh. This is a result from differences in CO_2_ concentrations (12.9 [warm] and 65.6 μM/kg [cold seawater]) and flow rates (5,710 [warm] and 2,580 kg/s [cold]).

### OTEC CO_2_ concentration

In the vacuum process, we assume all inert gases are removed from the water; however, CO_2_ is only removed to the extent as determined by Green and Guenther (1990). In the measurements reported by Sverdrup (1942), non-condensables in sea water are analyzed to contain 8 L/m^3^ oxygen and 11 L/m^3^ average nitrogen. Measurements on OTEC (Green and Guenther, 1990) show that 0.065 mol/m^3^ or 1.5 L/m^3^ CO_2_ is released from the cold water, with a total of 20.5 L/m^3^ non-condensables. The CO_2_ content of non-condensables is 7.3% at maximum if non-condensables from the warm and cold water stream are combined. If, however, the non-condensables from the cold stream are taken separately, the content of CO_2_ originated in deep cold water is higher at 14%. This follows from the ratio of 65 × 10^−3^ mol/m^3^ CO_2_, and the total concentration of non-condensables is 464 × 10^−3^ mol/m^3^ (Table 3). Normally, a compressor from the OTEC system extracts the non-condensables and the gases from the warm and cold water are mixed in one stream because there is no aim for harvesting CO_2_. This is also shown in Figure 1. The concentrations of the non-condensables emitted by cold and warm water differ because deep ocean water contains more CO_2_, and much less, if not negligible oxygen, as presented in Table 3.

**Table 3:** Calculated concentrations of non-condensables

Compound	Sea surface concentration			Deep sea layer concentration		
	(∗10^−3^ mol/m^3^)	(L/m^3^)	(%)	(∗10^−3^ mol/m^3^)	(L/m^3^)	(%)
Dissolved oxygen	200	4	23	20	0.4	4
Dissolved nitrogen	647	15	75	379	9	82
Dissolved CO_2_	13	0.2	1	65	1	14
Total	860	19	100	464	10	100

Deep sea layer generally is referred to as below 1000 meters of depth.

A higher CO_2_ content is desirable because further purification would require less energy and effort. If non-condensables would be extracted from the warm and cold water compartments in Figure 1 separately, the cold water non-condensable gas stream would contain a considerable higher concentration of carbon dioxide.

### CO_2_ purification from OC-OTEC

**Figure 2:**
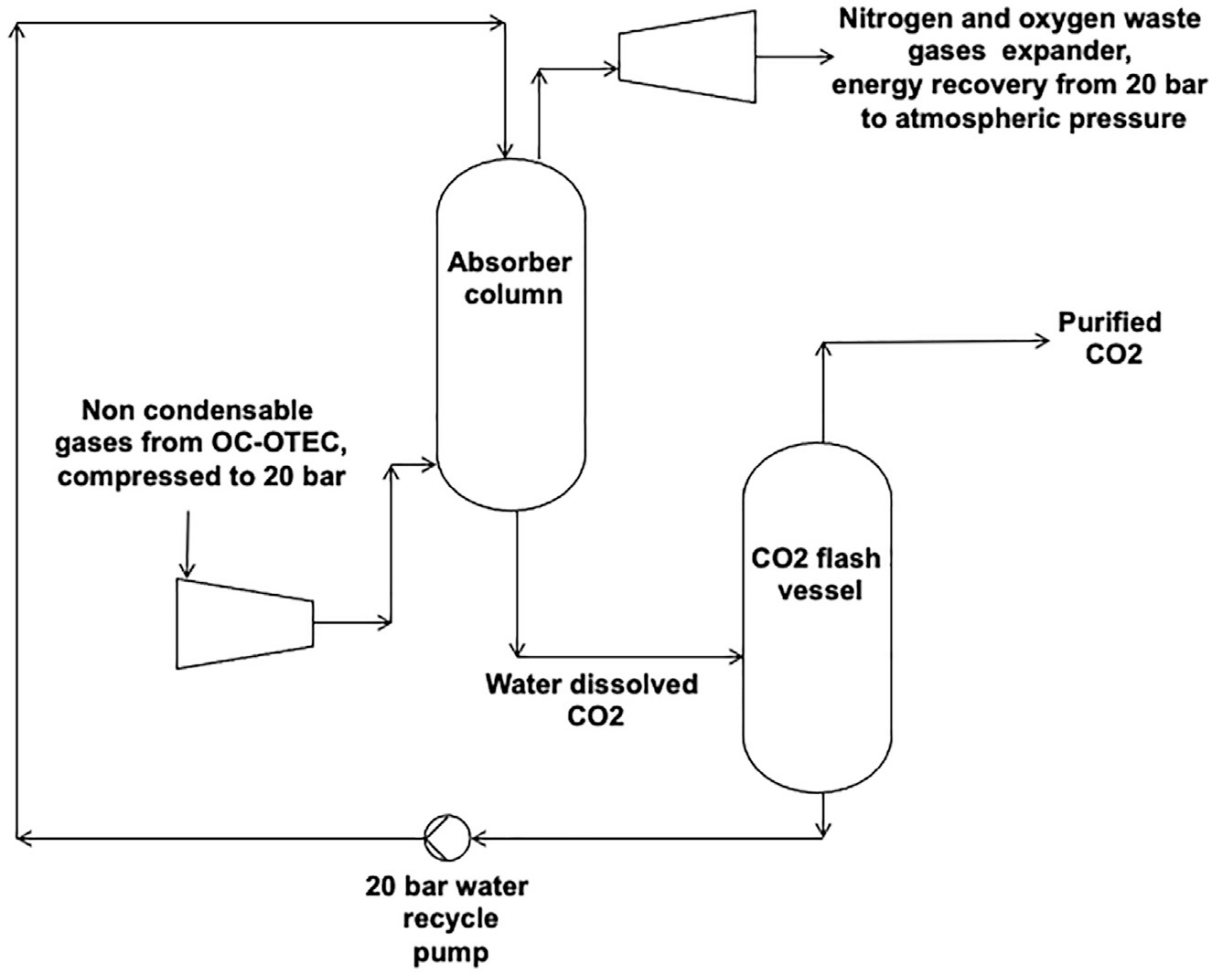
Process scheme of proposed CO_2_ from non-condensable purification unit This scheme is derived from a water absorption process that is often used to remove CO_2_ from raw methane gas that was produced in biogas installations. The modified process scheme differs from the biogas cleaning design by the fact that the raw gas is in this case non-condensables from OTEC instead of methane. The purification process is based on the difference in solubility in water of CO_2_ and nitrogen and oxygen. The gas mix is pressurized, and in a well-designed column, nearly all CO_2_ dissolves and a smaller part (1–5%) of also oxygen and nitrogen, as the solubility of nitrogen and oxygen is generally 1–5% (depending on temperature and pressure) of that of CO_2_ in water. When the water is depressurized, the dissolved CO_2_ and a small part of nitrogen and oxygen are “flashed” out of the recycle water.

When non-condensables from OC-OTEC are used directly, the CO_2_ content of the non-condensables is around 14% when extracted from deep ocean water (Table 3). If the degassing process of CO_2_ from non-condensables would be designed for widespread deployment, it should be optimized for CO_2_ concentration and energy usage. It is evident that from the calculations above the CO_2_ content in the non-condensables can be increased by extracting the water from a deep ocean layer to take water with low oxygen content. There is not necessarily more CO_2_, but less oxygen, that causes the CO_2_ percentage to be higher in deep ocean water than at the surface. Coming from an atmospheric CO_2_ concentration of 400 ppm to about 14% is a large concentration increase that was already delivered by OTEC. However, if the CO_2_ should be used, sold, or stored, it should be enriched. In order to enrich the carbon dioxide content, there are several technical options for purifying CO_2_ from gas streams (Sun et al., 2015; Bekkering et al., 2010). Similar technologies are widely applied at biogas installations and digester applications to separate methane from CO_2_. Figure 2 shows a schematic view of the modified design for such a CO_2_ water scrubber refining process.

This water scrubber type is used as a purification unit in biogas installations after digester units to be able to yield methane of higher purity (Seman and Harun, 2019). The working principle is the same as the ocean absorption of CO_2_ in water, where we note that CO_2_ has a much higher Henry solubility than other gases in water. The method in this paper contains no methane to be separated from CO_2_ but CO_2_ to be separated from nitrogen. The non-condensable gas mixture from the OC-OTEC is fed to the intake of a compressor that compresses the mixture to about 20 bar. The compressed non-condensables gas mixture is introduced in the absorber column, where pressurized sorbent water is injected at the top in counterflow with the gases. The CO_2_ is absorbed in the water, while the nitrogen and oxygen for a large part are not absorbed and exit the column at the top. The waste gases are then led over an expander to regain a large part of the compression energy of the mentioned compressor. The CO_2_ dissolved in the recycle water is depressurized in a flash vessel, where CO_2_ is flashed out of the recycle water as a result of depressurization. The recycle water is then again pumped to the absorber to take up new CO_2_. The compressor and expander are thermally coupled to each other; the compression heat of each compression stage is delivered to the expander stages in order to approach a reversible, isothermal compression for at least the nitrogen part. The non-condensable flow was derived using the concentrations in deep seawater from Table 3 and multiplied by the deep seawater flow from the mass balance from Vega (1999), which was 3.175 m^3^/s. Results are shown in Table 4.

**Table 4:** Molar flows of non-condensables for cold water flow of 3.175 m^3^/s

Compound	Deep sea layer CO_2_ concentration (mol/m^3^)	Molar flow of non condensables (mol/s)
Oxygen	0.02	0.064
Nitrogen	0.379	1.203
CO_2_	0.065	0.206
Total non condensables	0.464	1.473

The required compression and expansion work W is calculated as follows:

(Equation 6) W=∫PdV

We assumed the compression and expansion process to be isothermal because the compression heat is fed back to the expander. The average compression temperature is therefore assumed to be constant at ambient level. We calculate the required power to be able to estimate the investment, which is related to installed power for a type of compressor. We also calculate the power requirements to be able to derive the annual energy expenses.

The work W to compress the total molar flow n of 1.473 mol/s (Table 4) is calculated using the derivative of Equation 2 for isothermal compression:

(Equation 7) $$W=nRT\times ln\left(\frac{P_{1}}{P_{2}}\right)$$

Here, the start pressure P_1_ is atmospheric, and the end pressure P_2_ is 20 bar. R is the universal gas constant and T is the temperature of the compression, 293 K (ambient temperature). This yields W = 10.843 kJ/s for n = 1.47 mol/s. The same work excluding efficiency losses is theoretically delivered back by the expander. The total installed power for compressor/expander type equipment is then 2 times 10.843 = 21.686 kW.

The efficiencies of both expander and compressor are assumed to be 90%, and 14% of the CO_2_ containing non-condensable flow does not deliver back the compression energy. The losses are then 1.084 kW for the compressor and 1.084 kW for the expander, and 14% of 10.843 kW is 1.518 kW. All these losses taken together lead to an estimated net to be delivered power to the axle of 3.686 kW.

As a starting point, the mass and energy balance of the OC-OTEC plant as described by Vega (1999) was used. This plant produces 1.1 MW net electricity and needs about said 3.175 m^3^ cold water supply per second. As deep seawater contains said 65 mmol per m^3^, the CO_2_ flow in non-condensables then is 740 mol/hr.

Having a CO_2_ concentration of 14% in this non-condensable, the total non-condensable flow is 5,350 mol/hr. For such a flow of non-condensables in combination with a discharge pressure of 20 bar, a said 11 kW compressor is needed. So, for the purification of 750 mol/hr of CO_2_ in the non-condensable flow of 5,350 mol/hr, about said 3.7 kW is needed. The purity of delivered CO_2_ of these types of water scrubber systems is generally 80–90% (Sun et al., 2015). We calculated this yield purity in the STAR Methods section of this paper as well by using Henry constants. As calculated above, 750 mol of CO_2_ per hour is emitted from the cold water stream of the 1.1 MW OC-OTEC plant (Vega, 1999). This equals 33 kg per hour of CO_2_, leading to 0.11 kWh/kg. This is the CO_2_-specific energy requirement to drive this purification process. Bekkering et al. (2010) list 0.2 kWh/m^3^ for a water absorption scrubber. However, this is not a fully comparable technology because we use the expander in the bulk gas stream, which leads to much lower energy use. The energy cost per ton was calculated to be 5.5 euro if 50 euro per MWh for OTEC electricity is assumed. Banerjee et al., 2015 discuss an electricity price for OTEC on a 100 MW scale of 29 euro per MWh. If OTEC plants are built in future on such large scales, there is potential for cost improvement. Total annual CO_2_ yield at the scale of our calculation is 264 ton per year. This is based on the cold water flow of the OTEC mass balance of Vega (1999) and the deep sea CO_2_ content measured by Green and Guenther (1990). Our calculation is based on a 1 MW scale OC-OTEC. This CO_2_ purification method can also be applied as end of stack capture technology for industrial processes. The gas compositions of natural gas combustion originated flue gases and deep seawater non-condensibles are comparable. Individual cases may cause to vary concentrations and therefore capture costs may deviate.

### Economic analyses

The capital expenditures (capex) of the purification unit were estimated using the Lang factor cost estimation method (Lang, 1947a). The Lang factor is an average database factor of similar projects. The Lang factor formula is as follows:

(Equation 8) capex=∑Cprocess_eq×Fl

in which capex is the total project cost that equals the total process equipment cost (Cprocess_eq), multiplied by the Lang factor (Fl). For these types of simple processes and built as peripheral equipment, the factor 2.5 is common. This factor is a sum of subfactors described by Sinnot (2005). In this work, subfactors were defined for erection costs, piping, electrical, utilities, and buildings. The factor of 2.5 was obtained by adding the factors 1–6 and 10–13 (labor, fee, and contingency) in Table 6.1, Chapter 6 of Sinnot (2005). The estimation of capex is summarized in Table 5. The compressor price was obtained from a small-scale Makita 22 bar air compressor which was priced 755 euro for 1800 watts of installed power (Makita Compressor, 2021).

**Table 5:** Estimation of capex

Cost item	Price (euro)	Indicative price
Absorber column	500	1 m^3^ vessel 500 euro
Compressor	4337	400 euro/kW installed
Expander	4337	400 euro/kW installed
Pump	1000	1000 euro/kW installed
Expansion vessel	500	500 euro/m^3^
Total	10,674	–
Lang factor	2.5	–
Capex	26,685	–

**Figure 3:**
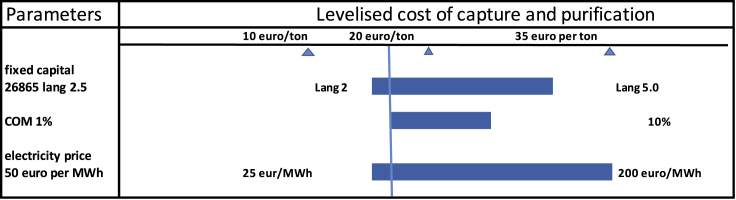
Sensitivity analyses of payback period of the carbon dioxide purification In the year 2025, ETS price is estimated at 50 euro/ton. PBP will decrease to 2.5 years in the year 2025, if CO_2_ price is following the projection. Fixed capital is varied +/− 50%, and CO_2_ price is varied to the projected price in ETS in the year 2030 and electricity price is varied between 30 and 100 euro per MWh.

We assume for the calculation that the produced CO_2_ is sold for the Emissions Trading Scheme (ETS) price, which is currently about 30 euro per ton (Markets Insider 2021). Electricity expense is 5.6 euro per ton, with an annual said yield of 264 ton. The annual revenue of this plant is calculated based on the profit per ton times the annual production, minus 1% of capital per year for operation and maintenance, leading to (30–5.6) x 264)-266.85 = 6,158 euro per year. Capex is 26,685 euro, so in a non-annualized approach, the simple economic payback period (PBP) is about 4.3 years. In Figure 3, a sensitivity analysis is shown which also provides insight into the payback when CO_2_ prices rise the coming years. It is expected that in 2030, ETS price would reach 100 euro per ton CO_2_. If by the year 2025 the price would be 50 euro per ton, a payback time of 2.5 years would be possible.

**Figure 4:**
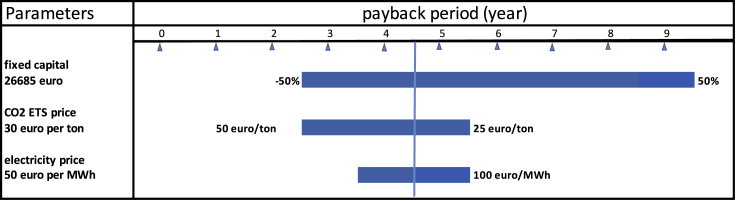
Sensitivity analyses of LCCP of the carbon dioxide purification based on parameters for a land-based plant Electricity prices vary from 25–200 euro per MWh, the Lang factor is varied from 2–5, and operation and maintenance are varied between 1 and 10% of capital per year.

Levelized carbon capture and purification costs (LCCP) for the OC-OTEC technology have been calculated by the following formula:

(Equation 9) $$LCCP=\frac{crf\times Cin+Com+Cpower}{CO_{2}net}$$

where crf is the capital recovery factor (equaling 0.11), using a 20-year depreciation period and an annual debt rate of 8% and an annual insurance rate of 1%, Cin is the capital investment of 26,685 euros, Com is the operation and maintenance cost (1% of capital per year), and Cpower is the annual electricity cost (OTEC electricity price of average 50 euro per MWh is assumed). Even if OTEC electricity is more expensive than expected, it is not that sensitive to the payback period like the capex for instance. Finally, CO_2_net is the produced amount in tons per year of purified CO_2_ from the OC-OTEC system. The levelized electricity cost of OTEC is assessed by Langer et al. (2020) to be in the range of 25–200 euro per MWh. The impact of this range on the LCCP is shown in Figure 4.

Levelized carbon capture and purification costs (annualized approach) were calculated based on these numbers to be 15–35 euro per ton. The accuracy of such a feasibility study using Lang factors is generally about 50% (Lang, 1947b). It is expected that due to economies of scale, this price will reduce further, while the ETS carbon price will likely rise.

### Conversion to carbon neutral synfuel

One of the examples of synthetic fuels is methanol, which can be made out of CO_2_ and hydrogen both originating from the ocean, with the use of OTEC electricity, and desalinated water coming also from OC-OTEC. Such a fuel could replace the need for fossil fuels and therewith arrest the accumulation of fossil CO_2_ in the atmosphere. Methanol can be stored in atmospheric pressure tanks, and combustion engines can run on it without much modification. The equation for the production of methanol is as follows:

(Equation 10) CO_2_ + 3H_2_→ CH_3_OH + H_2_O

In the above described OC-OTEC scenario, too less CO_2_ can be captured compared to the amount of hydrogen that is needed for methanol production. This is due to the fixed amount of cooling water. When methanol is made with this scenario, the surplus of electricity can be sold to the grid. The CO_2_ and hydrogen balance are calculated as follows. The enthalpy of combustion of hydrogen is 286 kJ/mol at a molar mass of 1 g/mol. At a megawatt scale, incorporating the efficiency of an electrolyzer of 75% at best (Kumar and Himabindu, 2019), 750 kW can be used to generate hydrogen. Based on enthalpy and installed power, 750/286 = 2.62 mol/s of hydrogen can be generated. For 1 mol of methanol, 3 mol hydrogen and 1 mol of CO_2_ are needed, referring to reaction (6). Thus, at 1 MW scale, a CO_2_ yield of 0.1675 mol/s is obtained, which will react with about 0.6 moles/s of hydrogen. For full utilization of the possibly generated hydrogen, 1/3 of 2.62 = 0.87 mol/s of CO_2_ is needed, which is 5.21 times the CO_2_ that is yielded in the unmodified scenario. For upgrading to equimolar CO_2_ yield to the produced hydrogen, 5.21 times the cold water flow is needed. The unmodified scenario could yield about 0.1675 mole of methanol/s on a 1 MW scale. An improved modified scenario with extra cooling water could hypothetically yield about 0.87 mole of methanol/s on a 1 MW scale.

In further research, we will search for an optimized configuration of an OC-OTEC system to produce more CO_2_ in relation to the amount of electricity. This could be done by partly bypassing the heat exchangers in the OTEC plant in the ratio as described above and return the discharge water all in all with much less pressure drop to save on parasitic (symbiotic) pumping cost. Future research will also include the economics of a synfuel production method that is using CO_2_ feedstock from OTEC byproduct. Terwel and Kerkhoven (2018) report a pilot demonstration of conversion from CO_2_ and hydrogen to kerosene via the Fischer-Tropsch reaction mechanism. This is also a promising conversion technology for non-fossil aviation, which could also benefit from the CO_2_ capture method proposed in this paper, in terms of scalability and low non-fossil CO_2_ feedstock price.

### Conclusions

In this paper, we have proposed a low-cost technology for indirect atmospheric capture of CO_2_. Current CO_2_ capture technologies are capturing CO_2_ from the air at a price of about 815–79 dollar per ton in the year 2020–2050, respectively. The proposed technology could contribute significantly to future CO_2_ capture technologies due to its scalability and low estimated costs of CO_2_ capture of 15–35 euro per ton in 2021. We described how atmospheric CO_2_ is captured by absorption in the ocean and subsequently from the ocean water by vacuum extraction in an OC-OTEC plant, being a source of captured CO_2_. We calculated the CO_2_ content to be 14% in non-condensable gas from cold water in an OC-OTEC plant. Thereafter, we performed a feasibility study on a slightly modified CO_2_ purification method using the same proven technology as commonly used for CO_2_ purification in biogas installations. Since the non-condensable gases from OTEC systems are regarded as a waste gas stream, we refer this stream as costless. However, we optimized the CO_2_ purification step on energy use and estimated capex. The energy use was calculated to be 21 kJ/mol CO_2_. This is more than an order of magnitude lower than CO_2_ removal energy required by water electrodialysis, which is 272 kJ/mol (Eisaman et al., 2012), where we note that this is nowadays one of the most promising indirect air capture methods. Based on an electricity price of 25–200 euro per MWh, opex-related CO_2_ capture costs are estimated to be less than 6 euro per ton. The levelized CO_2_ price was calculated to be 15–35 euro per ton in the year 2021.

Furthermore, the Fischer-Tropsch process can combine the CO_2_ with hydrogen to form kerosene (Terwel and Kerkhoven, 2018). The CO_2_ can also be directly used as a non-fossil fertilizer for agriculture and reforestation. For accelerated reforestation (a form of sequestration) and agriculture in remote dry areas with abundance of sunlight, the desalinated water stream from an OC-OTEC plant can be utilized along with the CO_2_ captured by the same plant.

### Limitations of study

In this paper, we focus on a coastal scenario (COM = 1%–10% of capital per year, 2.5 Lang factor [2–5 in sensitivity] with sensitivity results in Figure 4). In addition, the ratio of CO_2_ extracted is not in equilibrium with the required amount of hydrogen to make methanol. In fact, CO_2_ that is inherent to the amount of cold water is about 20% of the amount needed for equimolar CO_2_ when all electricity is converted to hydrogen for making methanol. The CO_2_ that is produced based on this study can be stored in a depleted gas field after compression and final oxygen removal or be used to make methanol fuel using electricity from OTEC. The CO_2_ that is converted to methanol is neutral in creditation; however, when stored, it can be credited as negative emission. Surplus of electricity can be sold. In this scenario as such (not optimized CO_2_ flow), the Gt per year scale is far away; however, in the current studied scenario, per megawatt installed power scale 0.2 kilotons per year can be captured. Looking at added sensitivity analyses for levelized carbon capture and purification cost, the range is in 15–35 euro per ton. Further, we like to note that unwanted downstream chemical and biological effects of CO_2_ extraction are avoided by a subsurface discharge of more than 50 m below sea level. Discharge water will be slightly less acidic due to proton acceptance in reaction 2 and 3 in section 2.2. It should be noted as well that in the estimation of the carbon capture price, we did not include costs for injection in depleted gas fields or alike since this depends very much on the specific situation.

In future work, adaptations to the design will be assessed to explore the possibility of equalizing the amount of hydrogen to the stoichiometric conditional CO_2_ amount that is necessary for methanol production. This way it may become possible to place the OTEC offshore on ideal locations to OTEC. For this, a higher water flow is needed, and this may impact the pumping losses and the OTEC electricity price. The effect on the OTEC design and methanol price is not assessed in this study but is subject to further research. Another motivation for adapting the flow is to be able to realistically come to Gt/year capture scale, as for every installed Gigawatt about 1080 kton (1 Megaton) of CO_2_ per year can be captured when the cold water flow is increased to the stoichiometric conditions for methanol production. On the other hand, coastal scenarios also may have a large advantage when lower electricity prices are originated in wind and solar OTEC hybrids for delivering hydrogen for later combination with indirect air captured CO_2_.

## STAR★Methods

### Key resources table

**Table:** 

REAGENT or RESOURCE	SOURCE	IDENTIFIER
**Deposited data**		

Measured concentrations of CO_2_ in seawater before and after applying OTEC-vacuum	Green and Guenther, 1990	N/A

### Resource availability

#### Lead contact

Further information should be directed to and will be fulfilled by the lead contact Wilfried van Sark (W.G.J.H.M.vanSark@uu.nl)

#### Materials availability

This study did not generate new unique materials.

#### Data and code availability

Data with analyses of seawater before and after OTEC are available from the paper of Green and Guenther (1990). This is publicly accessible.

### Method details

#### The OTEC non-condensable stream composition

This method is based on measurements as described above on seawater and concentrations of dissolved gases, before and after vacuum was applied to the water, as it fluxed through the vacuum compartments (condenser for cold water) of the OTEC installation.

Deep seawater CO_2_ concentration is 65 millimol/m^3^ lower after passing the OTEC (Green and Guenther, 1990). The non-condensables released, contain 7.3 grams (using 44 g/mole as molar weight of CO_2_) of CO_2_ per second at the megawatt scale when the concentration is multiplied by the cold water flow. Deep seawater dissolved gases are composed of 14% CO_2_ as calculated in this paper based on (Sverdrup et al., 1942) with the rest mostly nitrogen, as in the deep there is anaerobe dissimilation of organisms which uses oxygen and produce CO_2_. For purification, the non condensables are pressurized to 20 bar and are bubbling trough a water filled column (absorber) in counterflow with water. The purification process is based on the difference in solubility at different pressures and Henry's law related actual dissolving in water of CO_2_ and nitrogen and oxygen. The gas mix is pressurized and the following concentrations in water will be reached when gas and liquid phase reach equilibrium. The described technology can be classified as energy optimized Pressure Swing Absorption with a water sorbent (PSA). The system consists of an absorption step where the crude non condensables are pressurized and will dissolve in water under higher pressure and subsequently will flash out the water when pressure is released in the flash vessel.

#### Absorber calculation

In the absorber referring to Figure 2, pressure is 20 bar and concentrations are 14% v/v CO_2_ in the gas phase and Henry constant for CO_2_ is 0.034 L/mol bar and Henry constant for N_2_ is 0.00061 L/mol bar (Henry, 1803). When entering the absorber, the gases will dissolve into the sorbent water until it has reached equilibrium concentration at the pressure and temperature in the absorber. The concentration C of the individual gases in water in equilibrium with the gas phase is calculated using:

C = H x p

in which p is partial pressure of the gas in the gas phase, which is calculated with the gas fraction and actual pressure of the gas phase. For CO_2_ the gas fraction was 0.14, and when the pressure is raised to 20 bar the concentration in the sorbent in equilibrium with gas phase is calculated as follows.

C_CO2_ = H_CO2_ x 0.14 x 20 = 0.0952 mol CO_2_/liter (aq)

The concentration of nitrogen in the non condensables (0.86) is the remaining part in the non condensables, where we neglect the low oxygen concentration. The concentration in the water sorbent is calculated as follows at 20 bar in the absorber vessel.

C_N2_ = H_N2_ x 0.86 x 20 = 0.01049 mol N_2_/L (aq)

#### Flash vessel calculation

In the flash vessel in Figure 2, the pressure is decreased to atmospheric level of about 1 bar absolute pressure. The maximum solubility of the dissolved gases is lower when pressure in the absorber is decreased. The dissolved gases will therefore “flash” out of the water sorbent. The solubility of CO_2_ and nitrogen in water at 1 bar absolute pressure are 0.03 and 0.00007 mol/L respectively referring to physical properties in Engineering Toolbox (2021). In order to calculate the quantity of CO_2_ and nitrogen that evades the sorbent due to depressurization, we subtract the maximum dissolved concentration at 1 bar from the dissolved concentration at 20 bar. This process is the regeneration of the water sorbent and the sorbent will be reintroduced to the absorber afterward to take up new CO_2_. For every liter of sorbent that is entering the flash vessel, 0.01049–0.00007 = 0.01042 mol nitrogen and 0.0951–0.03 = 0.0652 mol CO_2_ flashes out of the vessel in the gas phase. Total mole composition is 0.0104 + 0.0652 = 0.0756 mol (g)/liter. The composition of CO_2_ in the exiting gas of the flash vessel is the CO_2_ concentration in the gas phase divided by the total amount of gas liberated from 1 L of sorbent. This is therefore a fraction of 0.0652/0.0756 = 0.86. Under the mentioned material balance conditions, we calculate the purity of the produced CO_2_ to be 86% CO_2_. In further research, column design should be elaborated on a possible optimization of exit CO_2_ concentration. This could be done by a recycle stream from the purified CO_2_ to the non-condensable gas compressor intake. This way the partial pressure of CO_2_ in the absorber is increased, so more CO_2_ will be dissolved in proportion to nitrogen. Hence, a higher purity yield of CO_2_ can be obtained. However, a reflux ratio also causes the non-condensables compressor to consume more electricity, which results in a higher CO_2_ price. Here an optimum should be sought on individual cases.
